# Respiratory support during neonatal and infant aeromedical interfacility transfers in the Western Cape, South Africa: a retrospective review

**DOI:** 10.1186/s12873-025-01403-9

**Published:** 2025-12-24

**Authors:** Andrit Lourens, Johanna Catharina Botha, Garth Moys, Cally Stephen, Nikita Werthmann, Jocelyn Park-Ross, Sandi Holgate

**Affiliations:** 1 Division of Emergency Medicine, Department of Family, Community and Emergency Medicine, Faculty of Health Sciences, University of Cape Town, Cape Town, South Africa; 2 Department of Clinical Health Sciences, School of Health Sciences, Faculty of Health, Natural Resources and Applied Sciences, Namibia University of Science and Technology, Windhoek, Namibia; 3 South African Red Cross Air Mercy Service, Cape Town, South Africa; 4 Division of Critical Care, Department of Anaesthesia and Perioperative Medicine, Faculty of Health Sciences, University of Cape Town, Cape Town, South Africa; 5 Department of Paediatrics and Child Health, Faculty of Medicine and Health Sciences, Stellenbosch University, Cape Town, South Africa

**Keywords:** nasal Continuous Positive Airway Pressure (nCPAP), Continuous Positive Airway Pressure [Mesh], Respiratory support, Neonates, Infants, Infant, Newborn [Mesh], Aeromedical, Interfacility transfers (IFTs)

## Abstract

**Background:**

Interfacility transfer (IFT) of neonates and infants is common in South Africa, with many needing respiratory support. Recently, interest in non-invasive ventilation, particularly nasal continuous positive airway pressure (nCPAP), during IFTs has increased; however, local evidence is limited. This study aimed to describe the characteristics of neonates and infants requiring respiratory support and to evaluate the introduction of nCPAP during IFTs by the South African Red Cross Air Mercy Service (AMS) in the Western Cape between 2017 and 2019.

**Methods:**

A retrospective descriptive review of all neonates (≤ 28 days) and infants ( > 28 days to ≤ 1 year) requiring respiratory support during AMS IFTs was conducted between 2017 and 2019.

**Results:**

Respiratory support was required for 70.4% (435/618) of all neonates and infants transported during the study period. Of the 435 IFTs, 61.4% (*n* = 267) were neonates and 51.9% (*n* = 224) males. Approximately two-thirds (*n* = 296, 68.0%) were transported by rotor-wing (RW) aircraft, and Emergency Care Practitioners (*n* = 344, 79.1%) were the most common primary crew member. The median stabilisation time for RW and fixed-wing (FW) aircraft IFTs was > 60 mins, with median RW mission times approximately 3 hrs and > 5.5 hrs for FW. Common diagnoses included respiratory distress syndrome in neonates and pneumonia in infants. During IFTs, 174 (40.0%) patients received oxygen (O_2_) therapy, 141 (32.4%) nCPAP, and 120 (27.6%) positive pressure ventilation, predominantly mechanical ventilation (*n* = 116, 26.6%). Neonates more commonly received nCPAP during IFTs and infants’ oxygen therapy (*p* < 0.001). Additionally, in neonates, the use of nCPAP increased over the three years, while O_2_ therapy declined (*p* < 0.001).

**Conclusion:**

This study highlights the frequency of neonatal and infant aeromedical IFTs requiring respiratory support and the increasing adoption of nCPAP during aeromedical IFTs in one South African province. The findings suggest that nCPAP is a feasible respiratory support modality in the aeromedical IFT context; however, the safe and effective implementation relies on careful patient selection, adequately trained personnel, and appropriate equipment. Further research is warranted to evaluate the overall safety and clinical outcomes of nCPAP during IFTs and develop robust protocols and guidelines tailored to the South African context.

**Supplementary information:**

The online version contains supplementary material available at 10.1186/s12873-025-01403-9.

## Background

Globally, under-five mortality remains high, with sub-Saharan Africa accounting for 57% of deaths [1]. Nearly half (47%) of the 4.9 million global under-five deaths in 2022 occurred during the neonatal period [1]. South Africa (SA) made progress in reducing infant mortality; however, neonatal mortality remained largely unchanged in recent times [2]. Despite the progress, many preventable fatalities continue to occur, in part due to limited access to essential, high-quality healthcare [1, 3].

Centralised specialist healthcare in SA and the shortage of critical care resources necessitate the interfacility transfer (IFT) of critically ill neonates and infants from district to regional or tertiary hospitals over long distances via ground and aeromedical transport [4–7]. A lack of transportation and delays in referral to higher levels of care, a shortage of neonatal intensive care beds, and inadequate equipment and facilities are key healthcare system challenges suggested to contribute to neonatal deaths [8, 9]. Although local evidence is limited, it highlights several challenges related to neonatal and infant IFTs, such as delays due to ambulance, paramedic, and equipment shortages, poor communication, and adverse events (AEs) [10–12]. Advanced Life Support (ALS) practitioners also report encountering poorly prepared patients, lack of clinical support, and pressures to conduct inappropriate transfers [11].

A key focus for neonatal and infant IFTs is the need to initiate or continue respiratory support [4, 6]. Respiratory distress syndrome (RDS), pneumonia, and meconium aspiration syndrome (MAS) are the most common reasons for neonatal IFTs [4, 5] whereas respiratory, gastrointestinal, and neurological problems are the most common causes in infants [13]. Historically, endotracheal intubation (ETI) and mechanical ventilation (MV) were the common strategy to support neonates with respiratory disease. However, as this strategy has been associated with the development of chronic lung disease, respiratory care is evolving in favour of Non-Invasive Ventilation (NIV), including Continuous Positive Airway Pressure (CPAP) and Humidified High-flow Nasal Cannula (HFNC) oxygen (O_2_) [14–16]. CPAP can reduce the need for exogenous surfactant administration and MV and minimise complications, risks, mortality, and hospital stay [14, 17, 18].

With the high demand for neonatal and infant IFTs across SA, including the Western Cape (WC) province [5–7], the associated challenges and the need for respiratory support, the integration of nasal CPAP (nCPAP) as an additional respiratory modality offers advantages and is gaining increasing interest. However, to minimise risks, the integration should be accompanied by a well-trained transfer team, suitable well-maintained equipment, and cautious patient selection [19–23].

In 2014, a private ambulance service introduced nCPAP initiation or continuation by ALS practitioners during neonatal IFTs in SA. The South African Red Cross Air Mercy Service (AMS) followed in 2016 with heated humidified nCPAP on both rotor-wing (RW) and fixed-wing (FW) aircraft [24, 25]. This study aimed to describe the characteristics of neonates and infants requiring respiratory support (O_2_ therapy via nasal cannula or face mask, nCPAP, or Positive Pressure Ventilation (PPV)) and to evaluate the introduction of nCPAP during IFTs by AMS in the WC between 2017 and 2019. The objectives were to describe the patient, transport, and clinical characteristics of neonates and infants requiring respiratory support and to compare the proportion of neonates and infants requiring respiratory support during AMS IFTs in the WC, SA, between 2017 and 2019.

## Methods

### Study design

A retrospective review of all neonates and infants requiring respiratory support during IFTs by AMS was conducted between January 1, 2017, and December 31, 2019.

### Study setting

The WC, one of nine SA provinces, is sub-divided into six districts; one large metropolitan area with a well-developed healthcare network, including tertiary and district hospitals (City of Cape Town (CPT)), and five districts (rural or peri-urban) separated by long distances and served by district or regional hospitals. Most of the WC is served by the government-operated Emergency Medical Service (EMS), while various private ambulance organisations serve the minority of the population with medical aid or insurance.

AMS is a non-profit organisation contracted to the WC Department of Health and Wellness to provide aeromedical and rescue services utilising Pilatus PC-12 (pressurised cabin) FW aircraft and Augusta 119 helicopters. AMS has two bases, in CPT Metropole (FW and RW aircraft) and Oudtshoorn (RW aircraft only) in the Eden district. RW aircraft land directly at healthcare facilities, whereas FW aircraft land at an airport, necessitating ground transport to and from referral and receiving healthcare facilities, which are generally facilitated by AMS crew or a ground EMS crew accompanied by the AMS crew. However, handovers to other ground EMS crews may occur on occasions when the ground crew is able to provide the same level of care.

The medical crew comprises at least two members, with the senior crew member most commonly a paramedic (Emergency Care Practitioner (ECP), National Diploma, or Critical Care Assistant paramedic) or a medical doctor. Legislation requires all crew members to be registered with the Health Professions Council of South Africa and to complete the Aviation for Health Care Providers Course. The Paediatric Advanced Life Support course is mandatory for senior medical crew. Training is supplemented by in-house and outsourced workshops and regular skills and simulation-based training.

AMS introduced heated humidified nCPAP [Westmed Neo-Pod™ T humidifier (RW) or Fischer Paykel MR850 humidifier (FW)] through short bi-nasal prongs or nasal masks delivered with Hamilton-T1, OxyMag or babyPAC 100™ transport ventilators in 2016 [25]. An evidence-based protocol was developed with neonatologists from Tygerberg Provincial Hospital (TBH), the largest tertiary hospital receiving patients, detailing the training process, a transfer checklist, and a reference sheet for clinical care. Medical crews were upskilled through a training program combining theoretical lectures, instructional videos and practical sessions. The training included equipment familiarisation and was reinforced by implementing quality assurance and feedback systems to support practitioners. A CPAP checklist, including a quick reference guide, is completed during nCPAP IFTs for quality assurance and training purposes.

### Study population and sampling

The study population included all neonates and infants requiring respiratory support (O_2_ therapy, nCPAP, or PPV) during AMS IFTs via RW and FW aircraft in the WC between January 1, 2017, and December 31, 2019. Neonates were defined as patients aged ≤ 28 days, whereas infants were defined as patients aged > 28 days to ≤ 1 year. O₂ therapy refers to the administration of supplemental oxygen using low-flow oxygen delivery devices, such as a nasal cannula or face mask, during the IFT, in contrast to the positive pressure respiratory support modalities of nCPAP and PPV.

### Data collection and management

Data were extracted from AMS data sources (electronic database, patient care reports, and CPAP checklists) into a Microsoft (MS) Excel spreadsheet [26]. AMS developed the electronic database in 2010, serving as a centralised system for record-keeping. AMS medical crew and operation centre staff manually enter data related to service requests, basic patient information and mission details. All data is stored on a secure server with oversight from the regional manager and the Information Technology service provider. Access to the database is password-protected and managed through a user interface.

The data extracted included incident information (mission date, base, client, and platform type, referral and receiving hospitals, mission times, and crew qualification), patient information (date of birth, age, weight, gender, diagnoses), clinical features, and treatment information (respiratory support and other treatments). All data points were reviewed for completeness, accuracy and duplicate entries. Additionally, patient care reports were used to verify the accuracy of database records.

A pilot study comprising approximately 10% of the intended target population selected randomly from the data sources for 2020 was conducted to evaluate the feasibility and practicalities of data collection, judge inclusion/exclusion criteria and assess data reliability [27]. The data collection form was revised, refined, and enhanced. Data access was limited to the researchers and AMS management. After capturing the data, the MS Excel spreadsheet was password-encrypted.

### Data analysis

Data were imported and analysed using IBM SPSS Statistics for Windows, Version 28 [28]. Normality was assessed using the Shapiro-Wilk Test, which indicated that numerical data were not normally distributed. Consequently, numerical data were summarised using median and interquartile range (IQR), while for categorical data, frequencies (counts and percentages) were reported. Data were presented in graphs and tables. Missing data points were excluded listwise (removing cases with at least one missing value in the analysed variable).

The Pearson Chi-Square test for Independence (χ^2^) was used to compare categorical variables. The independent variable was *patient group* (neonate and infant), or *year transported* (2017, 2018 and 2019), while the dependent variable was *respiratory support modality* (O_2_ therapy, nCPAP, or PPV). The effect size and magnitude of association between the variables were reported through Cramer’s V (φ_c_) (tables > 2x2) coefficients, as appropriate and judged per the ranges reported by Rea and Parker (1992, as cited in Sapra and Satish, 2021) [29]. Hypothesis tests were two-sided, and a p-value of < 0.05 was considered significant.

## Results

The dataset contained 460 entries, of which 25 (5.4%) were excluded due to incomplete data (*n* = 2, 0.4%) or duplicate case entries (*n* = 23, 5.0%), leaving 435 cases meeting the inclusion criteria. Our study included 70.4% (435/618) of all neonates and infants and 12.7% (435/3423) of all patients transported over the three years.

### Patient and transport characteristics

Of the included cases, 224 (51.9%) were male (3 missing data points), while 267 (61.4%) were neonates and 168 (38.6%) infants. A similar proportion of neonates and infants was transported per year, approximately two-thirds by RW (*n* = 296, 68.0%). CPT (58.8% vs 41.2%) and Oudtshoorn (66.2% vs 33.8%) bases transported more neonates than infants. The most common primary and secondary crew were ECPs (*n* = 344, 79.1%) and Emergency Care Technicians (ECTs) (*n* = 339, 77.1%), respectively (Table 1).

**Table 1:** Patient and transport characteristics

Characteristics	Neonates, n (%)	Infants, n (%)	Total, n (%)
**Sex**			
**Male**	139 (62.1%)	85 (37.9%)	224 (51.9%)
**Female**	127 (61.1%)	81 (38.9%)	208 (48.1%)
**Total**	**266 (61.8%)**	**166 (38.8%)**	**432 (100.0%) **^**a**^
**Year Transported**			
**2017**	95 (61.3%)	60 (38.7%)	155 (35.6%)
**2018**	80 (62.5%)	48 (37.5%)	128 (29.4%)
**2019**	92 (60.5%)	60 (39.5%)	152 (35.0%)
**Total**	**267 (61.4%)**	**168 (38.6%)**	**435 (100.0%)**
**Base Transported**			
**Cape Town**	167 (58.8%)	117 (41.2%)	284 (65.3%)
**Oudtshoorn**	100 (66.2%)	51 (33.8%)	151 (34.3%)
**Total**	**267 (61.4%)**	**168 (38.6%)**	**435 (100.0%)**
**Transport Platform**			
**Rotor Wing**	200 (67.6%)	96 (32.4%)	296 (68.0%)
**Fixed Wing**	67 (48.2%)	72 (51.8%)	139 (32.0%)
**Total**	**267 (61.4%)**	**168 (38.6%)**	**435 (100.0%)**
**Transport Mode**			
**In-arms**	21 (27.6%) ^b^	55 (72.4%)	76 (17.5%)
**Stretcher**	2 (2.9%)	67 (97.1%)	69 (15.9%)
**Incubator**	244 (84.1%)	46 (15.9%)	290 (66.7%)
**Total**	**267 (61.4%)**	**168 (38.6%)**	**435 (100.0%)**
**Primary Transport Crew**			
**CCA**	6 (54.5%)	5 (45.5%)	11 (2.5%)
**Ndip**	7 (50.0%)	7 (50.0%)	14 (3.2%)
**ECP**	223 (64.8%)	121 (35.2%)	344 (79.1%)
**Doctor**	31 (47.0%)	35 (53.0%)	66 (15.2%)
**Total**	**267 (61.4%)**	**168 (38.6%)**	**435 (100.0%)**
**Secondary Transport Crew**			
**AEA**	28 (66.7%)	14 (33.3%)	42 (9.9%)
**ECT**	220 (64.9%)	119 (35.1%)	339 (77.1%)
**CCA**	0 (0.0%)	3 (100.0%)	3 (0.7%)
**Ndip**	2 (33.3%)	4 (66.7%)	6 (1.8%)
**ECP**	14 (35.0%)	26 (65.0%)	40 (9.4%)
**Doctor**	3 (60.0%)	2 (40.0%)	5 (1.1%)
**Total**	**267 (61.4%)**	**168 (38.6%)**	**435 (100.0%)**

Footnote: ^a^ Three missing data points, ^b^ One transported with Kangaroo Mother Care

Abbreviations: AEA - Ambulance Emergency Assistant, CCA - Critical Care Assistant, ECP - Emergency Care Practitioner, ECT - Emergency Care Technician, NDip - National Diploma in Emergency Medical Care

The median age of neonates was 1 day (IQR = 0–6 days) and median weight 2000 g (IQR = 1300-3000 g). Gestational age was available for 63.7% (*n* = 170) of the neonates, of which the majority (*n* = 124, 72.9%) were preterm ( < 37 weeks). The median infant age was 3 months (IQR = 2–5 months), and the median weight 4.5 kg. (IQR = 3.1–6.2 kg)

Table 2 depicts median transport times per platform and patient group. Most RW mission times were shorter in comparison to FW, apart from ‘*Time from arrival at receiving facility/airport to IFT completion’*. The overall median stabilisation time was more than 60 mins for both platforms, whereas a slightly shorter median stabilisation time was noted for infant RW IFTs (56 mins, IQR 41–74 mins). The median transportation time for RW aircraft was 35 mins (IQR 22–48 mins) and 140 mins (IQR 124–158 mins) for FW aircraft, while the median total RW mission times were approximately 3 hrs and 5.5 hrs for FW.

**Table 2:** Transportation times per platform (rw or fw) and patient groups (neonates and infants)

Platform Type	Rotor Wing (RW)	Fixed Wing (FW)
**Neonates**	**Median (IQR) Time (mins), (n = 187)**	**Median (IQR) Time (mins), (n = 56)**^**b**^
Depart Base to arrive at Referral Facility	32 mins (24 - 42 mins)	84 mins (71 - 100 mins)
Arrive Referral Facility to Depart **(Stabilisation time)**	**65 mins (51 - 82 mins)**	**71 mins (46 - 98 mins)**
Transport Time (Referral to Receiving Facility)	33 mins (18 - 47 mins)	140 mins (126 - 158 mins)
Arrival Receiving Facility/Airport to IFT Completion	43 mins (33 - 53 mins)	31 mins (25 - 37 mins)
Total Mission Time	173 mins (151 - 211 mins)	333 mins (293 - 379 mins)
**Infants**	**Median (IQR) Time (mins), (n = 96)**	**Median (IQR) Time (mins), (n = 66)**^**b**^
Depart Base to arrive at Referral Facility	36 mins (27 - 43 mins)	85 mins (61 - 92 mins)
Arrive Referral Facility to Depart **(Stabilisation time)**	**56 mins (41 - 74 mins)**	**67 mins (49 - 87 mins)**
Transport Time (Referral to Receiving Facility)	38 mins (24 - 49 mins)	141 mins (121 - 161 mins)
Arrival Receiving Facility/Airport to IFT Completion	39 mins (30 - 50 mins)	26 mins (18 - 41 mins)
Total Mission Time	173 mins (142 - 202 mins)	322 mins (279 - 370 mins)
**Overall**	**Median (IQR) Time (mins), (n = 283)**^**a**^	**Median (IQR) Time (mins), (n = 122)**^**b**^
Depart Base to arrive at Referral Facility	33 mins (25 - 42 mins)	84 mins (67 - 97 mins)
Arrive Referral Facility to Depart **(Stabilisation time)**	**62 mins (46 - 79 mins)**	**68 mins (48 - 92 mins)**
Transport Time (Referral to Receiving Facility)	35 mins (22 - 48 mins)	140 mins (124 - 158 mins)
Arrival Receiving Facility/Airport to IFT Completion	42 mins (32 - 52 mins)	30 mins (21 - 40 mins)
Total Mission Time	173 mins (146 - 209 mins)	328 mins (283–371 mins)

Footnote: ^a^ 13 missing data points, all neonates, ^b^ 17 missing data points, 11 neonates and 6 infants. Missing data were excluded listwise (removing cases with at least one missing value in the analysed variable)

**Fig. 1:**
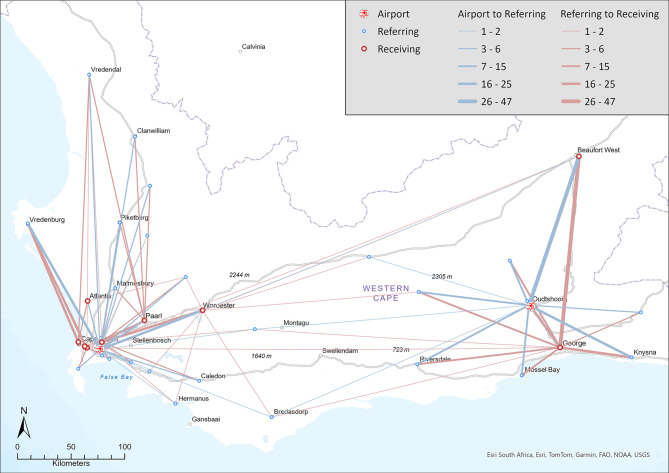
Map of rotor-wing interfacility transfers (UCT GIS Library)

**Fig. 2:**
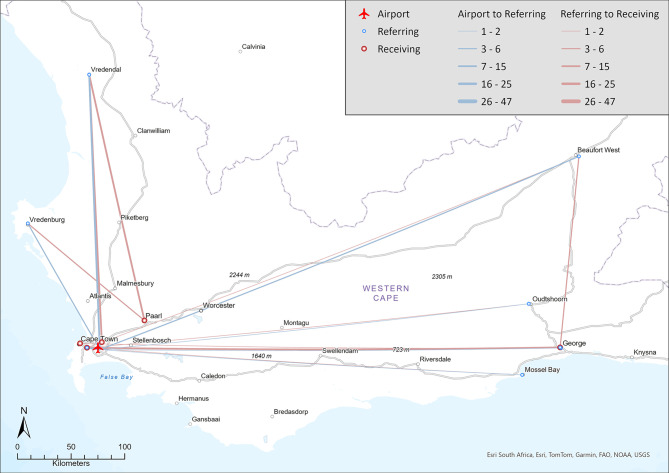
Map of fixed wing interfacility transfers (UCT GIS Library)

Additional File 1 details the number of IFTs per platform related to the referring and receiving facilities. Figure 1 depicts RW IFTs conducted per base, while Fig. 2 illustrates FW IFTs conducted across the WC.

As illustrated by Fig. 1, Vredenburg and Beaufort-West Provincial Hospitals were the most common referring facilities for the CPT and Oudtshoorn RW platforms, respectively, with TBH and George Provincial Hospital (GPH) the most common receiving facilities. GPH and Red Cross War Memorial Children’s Hospital were the most common referring and receiving facilities for FW aircraft (Fig. 2). Of the 139 FW IFTs, 13 (9.4%) were to or from healthcare facilities outside the WC (Gauteng, Northern and Eastern Cape).

### Clinical characteristics

The most common neonatal diagnoses were RDS (*n* = 119, 43.1%), sepsis (*n* = 45, 16.3%) and congenital heart defects (CHDs) (*n* = 43, 15.6%) and for infants, pneumonia (*n* = 64, 38.1%), respiratory distress/failure (*n* = 33, 19.6%), CHDs (*n* = 28, 16.7%) and sepsis (*n* = 26, 15.5%) (Additional File 2). Of all the patients, about half (*n* = 227, 52.2%) had two diagnoses and around 15.0% (*n* = 66, 15.2%) three.

Sixteen neonates received exogenous surfactant and 35 caffeine at the referring facility; eight received both. A gastric tube was placed in 190 (43.7%) and intravenous (IV) access in situ in 388 (89.2%) patients. Sixty-nine (15.9%) patients required vasopressor/inotropic infusions during IFTs, and two (one infant and one neonate) required two vasopressors/inotropes (dobutamine and adrenaline) (Table 3).

**Table 3:** Medication administration during IFTs

	Neonates, n (%)	Infants, n (%)	Total, n (%)
**Vasopressor/Inotropic Infusions**			
Adrenaline	7 (85.7%)	2 (14.3%)	9 (100.0%)
Dobutamine	41 (68.3%)	19 (31.7%)	60 (100.0%)
Dopamine	1 (50.0%)	1 (50.0%)	2 (100.0%)
**Other infusions during IFTs**			
Ketamine	5 (38.5%)	8 (61.5%)	13 (100.0%)
Midazolam	12 (46.2%)	14 (53.8%)	26 (100.0%)
Morphine	36 (61.0%)	23 (39.0%)	59 (100.0%)
**Other medication administered during IFTs**			
Adrenaline	1 (100.0%)	0 (0.0%)	1 (100.0%)
Adrenaline Nebulization	0 (0.0%)	1 (100.0%)	1 (100.0%)
Dextrose 5%	3 (75.0%)	1 (25.0%)	4 (100.0%)
Ketamine	8 (50.0%)	8 (50.0%)	16 (100.0%)
Opioids ^a^	7 (77.8%)	2 (22.2%)	9 (100.0%)
Prostaglandin	7 (100.0%)	0 (0.0%)	7 (100.0%)
Midazolam	3 (27.3%)	8 (72.7%)	11 (100.0%)
Rocuronium	3 (33.3%)	6 (66.7%)	9 (100.0%)

Footnote: ^a^ Including Fentanyl and Morphine

### Respiratory support on arrival and during AMS IFTs

Upon AMS arrival at the referring facility, 97.7% (*n* = 425) of patients were receiving respiratory support, with 2.3% (*n* = 7) on room air (3 missing data points). Table 4 illustrates the change (escalation or de-escalation) in respiratory support following AMS arrival and before the initiation of the IFT. The respiratory support for 70 (16.1%) patients was changed, 67 (15.4%) patients were escalated, and three (0.7%) were de-escalated. Of the 435 cases, 174 (40.0%) patients received O_2_ therapy during the IFT, 141 (32.4%) nCPAP and 120 (27.6%) PPV, 4 (1.0%) with Neopuff**®** via ETT and the remaining MV (*n* = 116, 26.6%) via a transport ventilator.

**Table 4:** Respiratory support escalation/de-escalation for transportation

On arrival at the referring facility	**Neonate, n (%)**^a^	Infant, **n (%)**^b^	Total, n (%)	Escalation/de-escalation for transportation	Neonate, n (%)	Infant, n (%)	Total, n (%)
**Room Air**	4 (1.5)	3 (1.8)	7 (1.6)	**O**^**2**^**Therapy**	4 (1.5)	3 (1.8)	7 (1.6)
**O**^**2**^**Therapy**	101 (38.1)	91 (54.4)	192 (44.3)	**nCPAP**	21 (7.9)	7 (4.1)	28 (6.4)
				**MV**	0 (0.0)	1 (0.6)	1 (0.2)
**HFNC O2**	2 (0.8)	0 (0.0)	2 (0.5)	**nCPAP**	2 (0.7)	0 (0.0)	2 (0.5)
**nCPAP**	80 (30.1)	33 (19.8)	113 (26.2)	**O**^**2**^**Therapy**	2 (0.7)	1 (0.6)	3 (0.7)
				**Neopuff® via ETT**	1 (0.4)	0 (0.0)	1 (0.2)
				**MV**	3 (1.1)	0 (0.0)	3 (0.7)
**Mask Neopuff®**	5 (1.9)	2 (1.2)	7 (1.6)	**nCPAP**	3 (1.1)	0 (0.0)	3 (0.7)
				**Neopuff® via ETT**	0 (0.0)	1 (0.6)	1 (0.2)
				**MV**	2 (0.7)	1 (0.6)	3 (0.7)
**Neopuff® via ETT**	11 (4.2)	1 (0.6)	12 (2.8)	**MV**	11 (4.1)	0 (0.0)	11 (2.5)
**BVMV via ETT**	4 (1.5)	2 (1.2)	6 (1.4)	**MV**	4 (1.5)	2 (1.2)	6 (1.4)
**MV**	58 (21.9)	35 (21.0)	93 (21.5)	**Neopuff® via ETT**	1 (0.4)	0 (0.0)	1 (0.2)
**Total**	**265 (61.3)**	**167 (38.7)**	**432 (100.0)**	**Total**	**54 (20.2)**	**16 (9.5)**	**70 (16.1)**

Footnote: ^a^ Two missing data points, both neonates were transported on nCPAP, ^b^ One missing data point, the infant was transported on O^2^ therapy Abbreviations: BVMR - Bag Valve Mask Reservoir, ETT - Endotracheal Tube, HFNC - High Flow Nasal Cannula, MV - Mechanical Ventilation, O^2^ - Oxygen

**Fig. 3:**
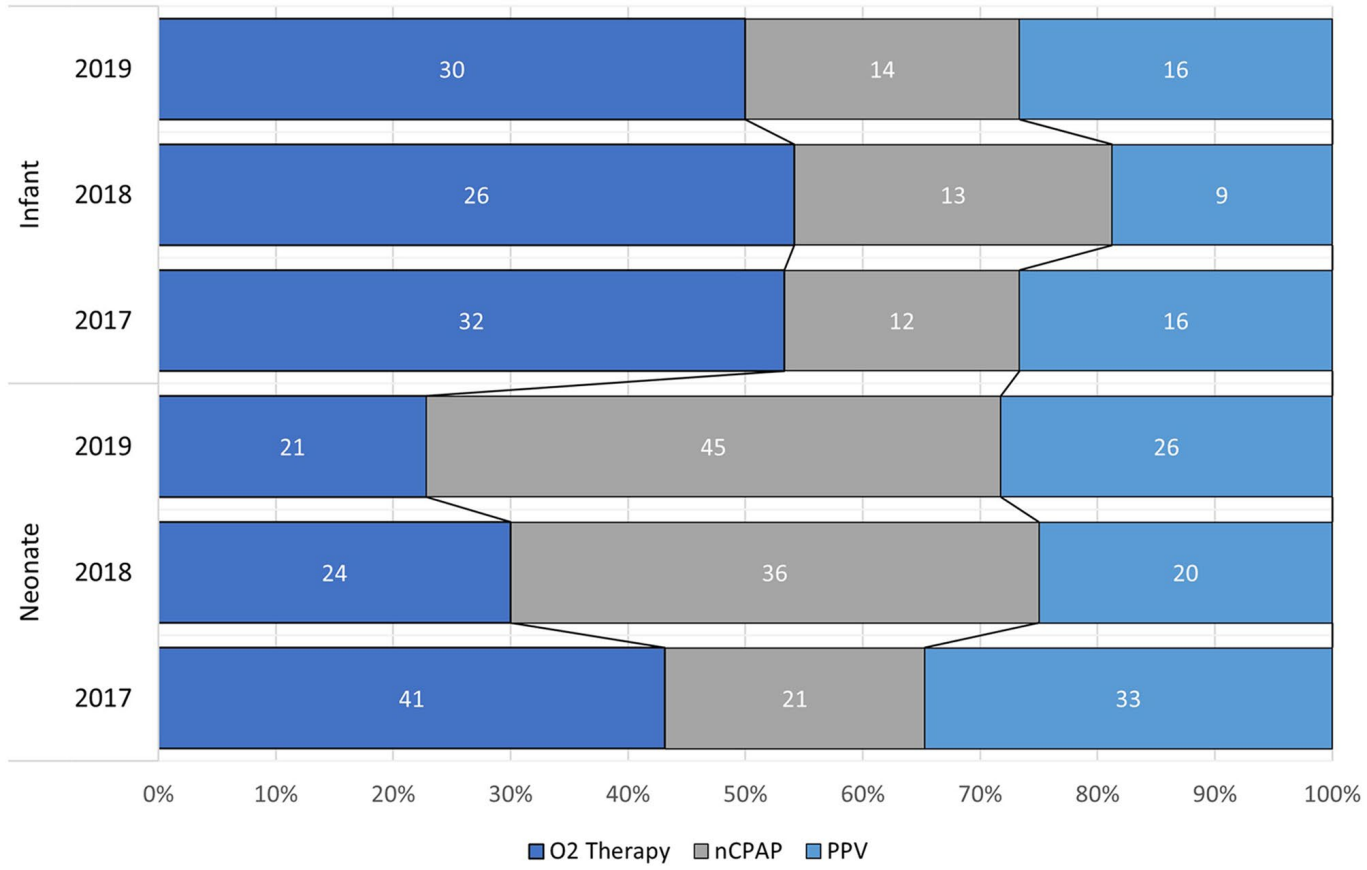
Respiratory support during ift for each year of transport for neonates and infants

Of the 267 neonates, 86 (32.2%) received O_2_ therapy via facemask or nasal prongs, 102 (38.2%) nCPAP and 79 (29.6%) PPV, 77 (28.8%) MV via a transport ventilator and 2 (0.8%) via a Neopuff during AMS IFT. For the 168 infants, 88 (52.4%) received O_2_ therapy via facemask or nasal prongs, 39 (23.2%) nCPAP and 41 (24.4%) PPV, 39 (23.2%) MV via a transport ventilator and 2 (1.2%) via a Neopuff during AMS IFT (Fig. 3).

Most patients received O_2_ therapy via nasal cannula (*n* = 170, 97.7%) with flow rates ranging between 0.5 to 6 litres per minute. Of the 141 patients transported on nCPAP, about a quarter (*n* = 35, 24.8%), mostly neonates (*n* = 28, 80%), were initiated by AMS crew, frequently in consultation with the receiving hospital. Using nasal prong nCPAP (*n* = 73, 67.0%) was more common than mask nCPAP (*n* = 36, 33.0%). At the referral facility, the median initial fraction of inspired oxygen (FiO_2_) was 45% (IQR = 35–70%), and the final (at handover) was 40% (IQR = 35–60%). The median initial (IQR = 5–5 cmH_2_O) and final nCPAP pressures (IQR = 5–5 cmH_2_O) were 5 cmH_2_O. Of the 120 (27.6%) patients who were intubated, 113 (95.0%) were initiated by the referring facility and six (5.0%, four neonates) by the AMS crew (one missing data point) at the referring facility.

A significant difference was found between the patient group (neonate and infant) and respiratory support modality (O_2_ therapy, nCPAP, or PPV) over the study period (χ^2^=18.640, df=2, *p* < 0.001) with a moderate strength of association (φ_c_ = 0.207) [29]. Over the study period, neonates more commonly received nCPAP (38.2% neonates versus 23.2% infants) and infants more commonly received O_2_ therapy (32.2% neonates and 52.4% infants). The proportion of neonates (29.6%) and infants (24.4%) transported with PPV remained similar.

For neonates, a significant difference was found between the year transported (2017, 2018, 2019) and the respiratory support modality (χ^2^ = 17.821, df = 4, *p* < 0.001) with a moderate strength of association (φ_c_ = 0.258) [29]. O_2_ therapy use in neonates declined (43.2% in 2017, 30.0% in 2018 and 22.8% in 2019) over the three years while nCPAP use increased (22.1% in 2017, 45.0% in 2018 and 48.9% in 2019), particularly between 2017 and 2018. PPV use remained relatively stable over the three years (34.7% in 2017, 25.0% in 2018 and 28.3% in 2019). No difference was observed between the respiratory support modality and the year (χ^2^ = 1.562, df = 4, *p* = 0.816) transported for infants.

## Discussion

This study provides a comprehensive overview of respiratory support during neonatal and infant IFTs by AMS in the WC, SA over three years and a review of the introduction of nCPAP after its implementation. The cohort of patients consisted predominantly of neonates with a similar proportion of males and females among the respective patient populations. Most patients were transported by RW, with neonates being transported more frequently by both WC bases. Neonates more commonly received nCPAP, whereas infants were more likely to receive O_2_ therapy. O_2_ therapy use in the neonate group declined, whereas the use of nCPAP increased over the three years after its implementation.

Our study highlights the frequent transportation of infants and neonates requiring respiratory support, with 70.4% of neonates and infants transported during aeromedical IFTs in the sample described requiring respiratory support. This finding is consistent with other SA studies showing high neonatal and infant transfer rates between healthcare facilities by EMS (ground and aeromedical transport) across the country [5, 6, 10]. Respiratory or pulmonary diseases and CHDs are common referral diagnoses for infants and neonates, particularly when requiring NIV [4–6, 19, 22]. Additionally, prematurity and infection, including sepsis, are other frequent diagnoses in SA studies [5, 10, 30]. According to Rhoda et al. [8] infection, prematurity, and congenital anomalies are among the leading causes of neonatal deaths in SA. These challenges are exacerbated by systemic issues such as limited resources and transportation barriers.

Consistent with our findings, Howard and Wenzel [6] found that regional and district facilities account for most neonatal and infant IFTs. More specifically, a regional facility accounted for most FW IFTs and district facilities for RW IFTs in the current study. This pattern suggests that most IFTs originated from peri-urban and rural locations where resources may be limited. The frequency of IFTs from peri-urban and rural areas is likely influenced by several factors such as patient acuity, facility capacity and the availability of specialised care. The choice of platform may also be influenced by factors such as the allocation of AMS resources and night landing capacity for FW across the province. The FW platform may likewise be preferred for IFTs over longer distances, particularly for further escalation to specialist care available only at tertiary-level hospitals.

The mean stabilisation and mission times observed in the current study were comparable to similar SA and international studies, while other studies suggest shorter times [6, 10, 31–34]. For instance, Howard and Wenzel (2014) conducted a study on paediatric ( < 13 years) aeromedical IFTs in the WC, SA, reporting similar mission times but shorter stabilisation times for both RW and FW platforms [6]. Venter et al. (2021) likewise reported shorter stabilisation time for critical care neonatal transfers in the private sector in SA (ground and aeromedical transport) [5]. The international study by Abdul Wahab et al. (2019) reported a mean stabilisation time of 113 (SD ± 52) mins for neonates not requiring any interventions before transfer and a significantly longer mean stabilisation time of 165 (SD ± 89) mins for neonates requiring at least 1 or more interventions [34]. In contrast, the study conducted in Switzerland by Leeman et al. (2020) reported a median stabilisation time of 50 (IQR 20–260) mins for neonatal aeromedical IFTs [32]. While the exact reasons for the median stabilisation time of > 60 minutes for both platforms in our study are unclear, some studies suggest that pre-transfer interventions such as IV access, surfactant administration, ETI and MV, etc., significantly prolonged stabilisation times [33, 34]. Additionally, the type and number of interventions [33, 34] and the patient acuity can likewise influence the duration of stabilisation. However, Abdul Wahab et al. (2019) do suggest that some stabilisation time was spent on tasks that were not essential, such as awaiting an ambulance or bed availability [34]. The FW platform in our study may likewise be delayed by the availability of a ground ambulance when transportation from the referring facility to the airport is required.

Most RW mission times were shorter than FW, apart from ‘*Time from arrival at receiving facility/airport to IFT completion’*. As previously highlighted, FW aircraft require additional ground transport between airports and healthcare facilities, which adds to the overall mission times. RW aircraft are also commonly utilised for short-distance IFTs, allowing for shorter transportation times compared to FW aircraft, which is utilised for longer distances [35]. This is also reflected in the current study.

nCPAP appears to be a feasible respiratory support modality during IFTs, particularly in the aeromedical setting; however, further research is required to comprehensively evaluate the overall safety and clinical outcomes in this context. Several international studies suggest that nCPAP can be safely and effectively implemented and utilised during neonate and infant IFTs [19–23]. The systematic review by Cheema et al. (2019) showed that 0.4% (3/858) of children (0–18 years) transported on NIV needed intubation or NIV escalation during IFTs, while between 1 and 4% of patients across the studies experienced AEs on NIV during IFTs, including desaturation, apnea, bradycardia, hypotension, BVMR, etc. [22]. Current evidence remains insufficient to draw definitive conclusions regarding the safety of nCPAP use during aeromedical IFTs in SA. Well-designed prospective studies are warranted to assess the overall safety and to determine the frequency and severity of AEs in this context.

Our results show an increased utilisation of nCPAP, particularly in neonates, over the study period, a finding also demonstrated by Resnick and Sokol [21]. This may suggest expanded access to and a growing confidence in using NIV in the prehospital setting. The benefits of nCPAP in reducing the mortality and morbidity of neonates and infants [17, 18, 23] support its broader implementation during IFTs of this patient population. However, its integration must be accompanied by meticulous patient selection (avoiding patients at risk of acute respiratory failure), a well-trained experienced IFT team, suitable well-maintained equipment [19–23] and continuous close monitoring in addition to ongoing education and good clinical governance, including robust protocols/guidelines [23]. Lategan et al. [36] estimated that the lack of CPAP availability in low- and middle-income countries contributes to a larger number of additional neonatal deaths. The advent of nCPAP during IFTs may assist in reducing these unnecessary deaths; however, further research is required to strengthen the understanding of utilising the modality during IFTs. Of interest would be determining the clinical condition at handover (receiving facilities) and the subsequent need for escalation to MV within 24 hours after the IFT. Resnick and Sokol [21] suggest that neonates requiring > 45–50% of O_2_ during IFTs were more likely to need MV at a later stage and thus may have benefited from MV at the time of the IFT. Further research may help substantiate this and aid in establishing stronger guidelines for nCPAP use and appropriate patient selection.

Our study findings underscore the critical need for effective and safe transportation of this vulnerable patient population. To achieve this, South African training institutions and EMS should prioritise comprehensive neonatal critical care training at the undergraduate and in-service levels, resource (including trained practitioners and appropriate well-maintained equipment) allocation and availability and robust protocols/guidelines to support decision-making. The ongoing need for transferring sick neonates and infants to higher levels of care necessitates such a focus, echoing the sentiments of other local authors in the field [4, 5, 10, 30].

Our study had several limitations. Though retrospective reviews of health records are a valuable and likely underutilised methodology, it is subject to incomplete/missing data, poorly recorded data and/or absent data since the data are not recorded specifically for research purposes [37]. Our study was, similarly, affected by data issues; however, to minimise the impact, we conducted a pilot study to determine the feasibility of data extraction and adjusted the data collection tool accordingly [27]. Due to the multiple AMS data sources, data extraction proved time-consuming and ensuring data accuracy was challenging. Moreover, several AMS clinical staff members assisted in the collection of data; however, to ensure data quality, a main data extractor from AMS monitored data collection throughout.

This is among the first studies to report on respiratory support, particularly nCPAP use, during IFTs for this population group in SA. These findings suggest that the use of nCPAP is feasible during neonatal and infant IFTs, provided the necessary equipment, trained personnel and clinical governance are in place. The data, however, were restricted to the WC; generalising the study findings beyond the province should be done cautiously. Our study did not aim to determine the clinical condition in which patients arrived at referring facilities, whether patient conditions improved or deteriorated during the out-of-hospital time, or to evaluate clinical outcomes. Although AEs were recorded in the patient records of IFTs, there was no standardised method for defining or capturing these events, nor interventions secondary to AEs. Routinely collected health data are known to be subject to potential underreporting, inconsistent definitions, and incomplete documentation, which may limit the accuracy and comparability of safety data derived from retrospective reviews. To accurately record these, well-designed prospective research is required as foregrounded.

## Conclusion

The transfer of neonates and infants requiring respiratory support is a critical and frequent occurrence in SA, demanding well-trained personnel, appropriate equipment, and robust clinical protocols/guidelines to ensure patient safety. The study findings indicate that nCPAP use during IFTs is feasible when supported by trained staff and effective systems. Growing confidence in nCPAP use during IFTs, alongside the potential benefits in reducing mortality and morbidity, suggests broader implementation could be advantageous, provided it is accompanied by rigorous training, continuous monitoring, and strong clinical governance to refine patient selection criteria and enhance clinical outcomes. However, prospective research is essential to explore the safety of nCPAP during IFTs, the impact of nCPAP on clinical conditions upon arrival at receiving facilities, patient outcomes post-transfer, and to establish stronger guidelines for respiratory support during neonatal and infant IFTs. Such research is crucial to strengthening the quality of care and improving outcomes for this vulnerable patient population, particularly in resource-limited settings such as SA.

## Electronic supplementary material

Below is the link to the electronic supplementary material.

Supplementary Material 1: Referring and Receiving Healthcare Facilities, Table displaying the frequency and percentage of cases per referring and receiving Healthcare Facilities

Supplementary Material 2: Patient Diagnosis, Table displaying the frequency and percentage of different diagnoses among the neonates and infants

## Data Availability

Data will be made available upon reasonable request and after consultation and upon agreement of AMS and Western Cape Department of Health and Wellness.

## Abbreviations

AE: Adverse Event
ALS: Advanced Life Support
AMS: South African Red Cross Air Mercy Service
CHD: Congenital Heart Defect
CPAP: Continuous Positive Airway Pressure
CPT: City of Cape Town
ECP: Emergency Care Practitioner
ECT: Emergency Care Technician
EMS: Emergency Medical Services
ETI: Endotracheal Intubation
FW: Fixed-wing
GPH: George Provincial Hospital
HFNC: Humidified High-flow Nasal Cannula
IFT: Interfacility Transfer
IV: Intravenous
IQR: Interquartile Range
MAS: Meconium Aspiration Syndrome
MS: Microsoft
MV: Mechanical Ventilation
nCPAP: nasal Continuous Positive Airway Pressure
NIV: Non-Invasive Ventilation
O^2^: Oxygen
PPV: Positive Pressure Ventilation
RDS: Respiratory Distress Syndrome
RW: Rotor-wing
TBH: Tygerberg Provincial Hospital
SA: South Africa
WC: Western Cape
