# Supplementary material for: Respiratory support during neonatal and infant aeromedical interfacility transfers in the Western Cape, South Africa: a retrospective review
Source: BMC Emerg Med. 2025 Dec 24;25:258. doi: 10.1186/s12873-025-01403-9 (PMC12728987; doi:10.1186/s12873-025-01403-9)
Supplement: Supplementary file 1 — Supplementary Material 1: Referring and Receiving Healthcare Facilities, Table displaying the frequency and percentage of cases per referring and receiving Healthcare Facilities [file 12873_2025_1403_MOESM1_ESM.docx]

**Additional File 1:** Referring and Receiving Healthcare Facilities

| **Referring Healthcare Facilities** | | | |
| --- | --- | --- | --- |
| **Healthcare Facilities** | **RW, n (%)** | **FW, n (%)** | **Total, n (%)** |
| Alan Blyth Hospital | 12 (4.1%) | 0 (0.0%) | 12 (2.8%) |
| **Beaufort-West Provincial Hospital** | **49 (16.6%)** | 4 (2.9%) | 53 (12.2%) |
| Caledon Provincial Hospital | 3 (1.0%) | 0 (0.0%) | 3 (0.7%) |
| Ceres Provincial Hospital | 6 (2.0%) | 0 (0.0%) | 6 (1.4%) |
| Clanwilliam Hospital | 7 (2.4%) | 0 (0.0%) | 7 (1.6%) |
| Citrusdal Provincial Hospital | 5 (1.7%) | 0 (0.0%) | 5 (1.2%) |
| False Bay Hospital | 3 (0.7%) | 0 (0.0%) | 3 (0.7%) |
| Frere Hospital (Eastern Cape) | 0 (0.0%) | 6 (1.4%) | 6 (1.4%) |
| **George Provincial Hospital** | 2 (0.7%) | **79 (57.7%)** | 81 (18.7%) |
| George Medi Clinic | 0 (0.0%) | 3 (2.2%) | 3 (0.7%) |
| Grabouw CDC | 1 (0.3%) | 0 (0.0%) | 1 (0.2%) |
| Helderberg Provincial Hospital | 3 (1.0%) | 0 (0.0%) | 3 (0.7%) |
| Hermanus Provincial Hospital | 3 (1.0%) | 0 (0.0%) | 3 (0.7%) |
| Khayelitsha Provincial Hospital | 4 (1.4%) | 0 (0.0%) | 4 (0.9%) |
| Knysna Provincial Hospital | 22 (7.4%) | 0 (0.0%) | 22 (5.1%) |
| Laingsburg Provincial Hospital | 1 (0.3%) | 0 (0.0%) | 1 (0.2%) |
| Life Bay View Hospital | 0 (0.0%) | 1 (0.7%) | 1 (0.2%) |
| Mosselbay Provincial Hospital | 10 (3.4%) | 0 (0.0%) | 10 (2.3%) |
| Mitchells Plain District Hospital | 1 (0.3%) | 0 (0.0%) | 1 (0.2%) |
| Oudtshoorn Provincial Hospital | 25 (8.4%) | 1 (0.7%) | 26 (6.0%) |
| Otto du Plessis Hospital | 3 (1.0%) | 0 (0.0%) | 3 (0.7%) |
| Paarl Provincial Hospital | 12 (4.1%) | 0 (0.0%) | 12 (2.8%) |
| Porterville Provincial Hospital | 1 (0.3%) | 0 (0.0%) | 1 (0.2%) |
| Prince Albert Provincial Hospital | 10 (3.4%) | 0 (0.0%) | 10 (2.3%) |
| Radie Kotze Hospital | 3 (1.0%) | 0 (0.0%) | 3 (0.7%) |
| Red Cross War Memorial Children's Hospital | 0 (0.0%) | 18 (13.1%) | 18 (4.2%) |
| Robertson Provincial Hospital | 2 (0.7%) | 0 (0.0%) | 2 (0.5%) |
| Riversdale Provincial Hospital | 15 (5.1%) | 0 (0.0%) | 15 (3.5%) |
| Springbok Hospital (Northern Cape) | 0 (0.0%) | 1 (0.7%) | 1 (0.2%) |
| Swartland Hospital | 6 (2.0%) | 0 (0.0%) | 6 (1.4%) |
| Uniondale Provincial Hospital | 3 (1.0%) | 0 (0.0%) | 3 (0.7%) |
| **Vredenburg Hospital** | **43 (14.5%)** | 4 (1.9%) | 47 (10.9%) |
| Vredendal Hospital | 12 (4.1%) | 19 (13.9%) | 31 (7.2%) |
| Wesfleur Provincial Hospital | 3 (1.0%) | 0 (0.0%) | 3 (0.7%) |
| Wits Donald Gordon Medical Centre (Gauteng) | 0 (0.0%) | 1 (0.7%) | 1 (0.2%) |
| Worcester Provincial Hospital | 26 (8.8%) | 0 (0.0%) | 26 (6.0%) |
| **Total** | **296 (68.4)** | **137** ^a^ **(31.6%)** | **433 (100.0%)** |
| **Receiving Healthcare Facilities** | | | |
| Beacon Bay (Eastern Cape) | 1 (0.3%) | 0 (0.0%) | 1 (0.2%) |
| Beaufort-West Provincial Hospital | 1 (0.3%) | 0 (0.0%) | 1 (0.2%) |
| Christiaan Barnard Memorial Hospital | 0 (0.0%) | 2 (1.4%) | 2 (0.5%) |
| Frere Hospital (Eastern Cape) | 0 (0.0%) | 4 (2.9%) | 4 (0.9%) |
| **George Provincial Hospital** | **146 (49.3%)** | 16 (11.5%) | 162 (37.2%) |
| Groote Schuur Provincial Hospital | 10 (3.4%) | 0 (0.0%) | 10 (2.3%) |
| Mowbray Provincial Hospital | 1 (0.3%) | 0 (0.0%) | 1 (0.2%) |
| Netcare Blaauwberg Hospital | 0 (0.0%) | 1 (0.7%) | 1 (0.2%) |
| New Somerset Provincial Hospital | 31 (10.5%) | 0 (0.0%) | 31 (7.1%) |
| Paarl Provincial Hospital | 22 (7.4%) | 12 (8.6%) | 34 (7.8%) |
| **Red Cross War Memorial Children's Hospital** | 24 (8.1%) | **92 (66.2%)** | 116 (26.7%) |
| Tygerberg Provincial Hospital | 52 (17.6%) | 11 (7.9%) | 63 (14.5%) |
| Wesfleur Provincial Hospital | 1 (0.3%) | 0 (0.0%) | 1 (0.2%) |
| Worcester Provincial Hospital | 8 (2.7%) | 0 (0.0%) | 8 (1.8%) |
| **Total** | **296 (68.1%)** | **139 (31.9%)** | **435 (100.0%)** |
| **Footnote:** ^a^ Two missing data points | | | |

- Additional File 1, Microsoft Word (.docx), Referring and Receiving Healthcare Facilities, Table displaying the frequency and percentage of cases per referring and receiving Healthcare Facilities.
