# Supplementary material for: Respiratory support during neonatal and infant aeromedical interfacility transfers in the Western Cape, South Africa: a retrospective review
Source: BMC Emerg Med. 2025 Dec 24;25:258. doi: 10.1186/s12873-025-01403-9 (PMC12728987; doi:10.1186/s12873-025-01403-9)
Supplement: Supplementary file 2 — Supplementary Material 2: Patient Diagnosis, Table displaying the frequency and percentage of different diagnoses among the neonates and infants [file 12873_2025_1403_MOESM2_ESM.docx]

**Additional File 2:** Patient Diagnosis

| **Diagnosis** | **Neonates, n (%)** | **Infants, n (%)** | **Total, n (%)** |
| --- | --- | --- | --- |
| **General** | | | |
| Amniotic Band Syndrome | 1 (0.9%) | 0 (0.0%) | 1 (0.6%) |
| Anaemia | 0 (0.0%) | 2 (3.4%) | 2 (1.2%) |
| APERT Syndrome | 2 (1.7%) | 0 (0.0%) | 2 (1.2%) |
| Cellulitis | 0 (0.0%) | 1 (1.7%) | 1 (0.6%) |
| Complete Wound Dehiscence | 0 (0.0%) | 1 (1.7%) | 1 (0.6%) |
| Congenital Syphilis | 4 (3.4%) | 2 (3.4%) | 6 (3.4%) |
| Dehydration | 0 (0.0%) | 2 (3.4%) | 2 (1.2%) |
| Dysmorphic Features | 2 (1.7%) | 1 (1.7%) | 3 (1.7%) |
| Disseminated TB | 0 (0.0%) | 3 (5.1%) | 3 (1.7%) |
| Failure to Thrive | 0 (0.0%) | 1 (1.7%) | 1 (0.6%) |
| Fetal Alcohol Syndrome | 0 (0.0%) | 1 (1.7%) | 1 (0.6%) |
| Haemolytic Uremic Syndrome | 0 (0.0%) | 1 (1.7%) | 1 (0.6%) |
| Hypo/Hyperglycaemia | 8 (6.8%) | 1 (1.7%) | 9 (5.2%) |
| Hypernatremia | 0 (0.0%) | 1 (1.7%) | 1 (0.6%) |
| Hyperthyroidism | 0 (0.0%) | 1 (1.7%) | 1 (0.6%) |
| Hypothermia | 1 (0.9%) | 0 (0.0%) | 1 (0.6%) |
| Intra Uterine Growth Restriction | 1 (0.9%) | 0 (0.0%) | 1 (0.6%) |
| Jaundice | 6 (5.1%)^a^ | 1 (1.7%) | 7 (4.0%) |
| Kwashiorkor | 0 (0.0%) | 1 (1.7%) | 1 (100.0%) |
| Low Birth Weight | 21 (17.9%) | 2 (3.4%) | 23 (13.1%) |
| Metabolic Acidosis | 1 (0.9%) | 5 (8.5%) | 6 (3.4%) |
| Multi-Organ Failure | 0 (0.0%) | 1 (1.7%) | 1 (0.6%) |
| Nosocomial Infection | 1 (0.9%) | 0 (0.0%) | 1 (0.6%) |
| Premature | 24 (20.5%) | 1 (1.7%) | 25 (14.2%) |
| Sepsis | 45 (38.4%) | 26 (44.1%) | 71 (40.4%) |
| TB Adenopathy | 0 (0.0%) | 1 (1.7%) | 1 (0.6%) |
| Trisomy 21 | 0 (0.0%) | 2 (3.4%) | 2 (1.2%) |
| Wound Sepsis | 0 (0.0%) | 1 (1.7%) | 1 (0.6%) |
| **Total** | **117 (66.5%)** | **59 (33.5%)** | **176 (100.0%)** |
| **Respiratory** | | | |
| Atelectasis | 0 (0.0%) | 1 (0.7%) | 1 (0.3%) |
| Apnoea | 10 (4.9%) | 3 (2.2%) | 13 (3.7%) |
| ARDS | 0 (0.0%) | 4 (2.8%) | 4 (0.9%) |
| Aspiration | 1 (0.5%) | 1 (0.7%) | 2 (0.5%) |
| Bronchitis | 0 (0.0%) | 5 (3.5%) | 5 (1.1%) |
| Bronchiolitis | 0 (0.0%) | 6 (4.2%) | 6 (1.4%) |
| Choanal Atresia | 5 (2.4%) | 0 (0.0%) | 5 (1.1%) |
| Congenital Diaphragmatic Hernia | 1 (0.5%) | 0 (0.0%) | 1 (0.3%) |
| Congenital Laryngeal Stridor | 1 (0.5%) | 0 (0.0%) | 1 (0.3%) |
| Congenital Lung Disease | 2 (1.0%) | 0 (0.0%) | 2 (0.5%) |
| Croup | 0 (0.0%) | 1 (0.7%) | 1 (0.3%) |
| Hypoxia | 1 (0.5%) | 0 (0.0%) | 1 (0.3%) |
| Lung Cyst | 0 (0.0%) | 1 (0.7%) | 1 (0.3%) |
| Lower Respiratory Tract Infection | 1 (0.5%) | 5 (3.5%) | 6 (1.4%) |
| Meconium Aspiration Syndrome | 24 (11.7%) | 0 (0.0%) | 24 (5.9%) |
| Obstructive Breathing | 0 (0.0%) | 1 (0.7%) | 1 (0.3%) |
| Other Pulmonary Heart Diseases | 0 (0.0%) | 1 (0.7%) | 1 (0.3%) |
| Persistent Pulmonary Hypertension of the Newborn | 3 (1.5%) | 0 (0.0%) | 3 (0.7%) |
| Pleural Effusion | 1 (0.5%) | 0 (0.0%) | 1 (0.3%) |
| Pneumonia | 24 (11.7%) | 64 (45.1%) | 88 (25.3%) |
| Pneumothorax | 1 (0.5%) | 2 (1.4%) | 3 (0.7%) |
| Pulmonary Haemorrhage | 2 (1.0%) | 0 (0.0%) | 2 (0.5%) |
| Pulmonary Hypertension | 3 (1.5%) | 2 (1.4%) | 5 (1.1%) |
| Pulmonary Oedema | 0 (0.0%) | 1 (0.7%) | 1 (0.3%) |
| Pulmonary Tuberculosis | 0 (0.0%) | 7 (4.9%) | 7 (1.6%) |
| Pulmonary Shunt | 0 (0.0%) | 1 (0.7%) | 1 (0.3%) |
| Respiratory Distress/Failure | 3 (1.5%) | 33 (23.2%) | 36 (10.4%) |
| Respiratory Distress Syndrome | 119 (57.8%) | 0 (0.0%) | 119 (34.2%) |
| Transient Tachypnoea of Newborn | 1 (0.5%) | 0 (0.0%) | 1 (0.3%) |
| Tracheal Stenosis | 0 (0.0%) | 1 (0.7%) | 1 (0.3%) |
| Upper Airway Obstruction | 3 (1.5%) | 0 (0.0%) | 3 (0.7%) |
| Upper Respiratory Tract Infection | 0 (0.0%) | 2 (1.4%) | 2 (0.5%) |
| **Total** | **206 (59.2%)** | **142 (40.8%)** | **348 (100.0%)** |
| **Cardiovascular** | | | |
| Cardiac Failure | 1 (2.1%) | 6 (13.3%) | 7 (7.5%) |
| Cardiac Murmur | 0 (0.0%) | 1 (2.2%) | 1 (1.1%) |
| Cardiomegaly | 1 (2.1%) | 2 (4.4%) | 1 (1.1%) |
| Complete Heart Block | 1 (2.1%) | 0 (0.0%) | 1 (1.1%) |
| Congenital Heart Defect | 43 (89.6%) | 28 (62.2%) | 71 (76.3%) |
| Dextrocardia | 1 (2.1%) | 1 (2.2%) | 2 (2.2%) |
| Dilated Cardiomyopathy | 0 (0.0%) | 1 (2.2%) | 1 (1.1%) |
| Infective endocarditis | 0 (0.0%) | 1 (2.2%) | 1 (1.1%) |
| Pacemaker Failure | 0 (0.0%) | 1 (2.2%) | 1 (1.1%) |
| Pericardial Effusion | 0 (0.0%) | 2 (4.4%) | 2 (2.2%) |
| Post Cardiac Arrest | 0 (0.0%) | 1 (2.2%) | 1 (1.1%) |
| Shock (all including Cardiogenic Shock) | 1 (2.1%)^b^ | 1 (2.2%) | 2 (2.2%) |
| **Total** | **48 (51.6%)** | **45 (48.4%)** | **93 (100.0%)** |
| **Neurological** | | | |
| Arteriovenous Aneurysm - Congenital Cerebral Infarction | 1 (2.5%) | 0 (0.0%) | 1 (1.5%) |
| Brain Death | 1 (2.5%) | 0 (0.0%) | 1 (1.5%) |
| Congenital Hypotonia | 0 (0.0%) | 1 (3.6%) | 1 (1.5%) |
| Hydrocephalus | 0 (0.0%) | 7 (25.0%) | 7 (10.3%) |
| Hypoxic-Ischemic Encephalopathy | 29 (72.5%) | 3 (10.7%) | 32 (47.1%) |
| Intraventricular haemorrhage | 0 (0.0%) | 1 (3.6%) | 1 (1.5%) |
| Meningitis | 0 (0.0%) | 2 (7.1%) | 2 (3.0%) |
| Traumatic Brain Injury | 0 (0.0%) | 1 (3.6%) | 1 (1.5%) |
| TB Meningitis | 0 (0.0%) | 4 (14.3%) | 4 (5.9%) |
| Seizures | 4 (10.0%) | 6 (21.4%)^c^ | 10 (14.7%) |
| Severe Brain Atrophy | 0 (0.0%) | 1 (3.6%) | 1 (1.5%) |
| Spina Bifida | 4 (10.0%) | 0 (0.0%) | 4 (5.9%) |
| Subdural Haemorrhage | 1 (2.5%) | 1 (3.6%) | 2 (3.0%) |
| Ventriculitis | 0 (0.0%) | 1 (3.6%) | 1 (1.5%) |
| **Total** | **40 (58.8%)** | **28 (41.2%)** | **68 (100.0%)** |
| **Abdominal** | | | |
| Acute Gastroenteritis | 0 (0.0%) | 5 (31.3%) | 5 (11.9%) |
| Bowel Obstruction | 6 (23.1%) | 2 (12.5%) | 8 (19.1%) |
| Bowel Perforation | 1 (3.8%) | 0 (0.0%) | 1 (2.4%) |
| Congenital Haemochromatosis | 1 (3.8%) | 0 (0.0%) | 1 (2.4%) |
| Congenital Nephrotic Syndrome | 1 (3.8%) | 0 (0.0%) | 1 (2.4%) |
| Congenital Renal Cyst | 1 (3.8%) | 0 (0.0%) | 1 (2.4%) |
| Duodenal Atresia | 1 (3.8%) | 0 (0.0%) | 1 (2.4%) |
| Gastrointestinal Tract Bleed | 1 (3.8%) | 1 (6.3%) | 2 (4.8%) |
| Gastroschisis | 1 (3.8%) | 0 (0.0%) | 1 (2.4%) |
| Hydronephrosis | 2 (7.7%) | 0 (0.0%) | 2 (4.8%) |
| Inguinal hernia | 0 (0.0%) | 1 (6.3%) | 1 (2.4%) |
| Intussusception | 0 (0.0%) | 1 (6.3%) | 1 (2.4%) |
| Liver Mass | 0 (0.0%) | 1 (6.3%) | 1 (2.4%) |
| Necrotising Enterocolitis | 8 (30.8%) | 0 (0.0%) | 8 (19.1%) |
| Obstructive Uropathy | 1 (3.8%) | 0 (0.0%) | 1 (2.4%) |
| Oesophageal Atresia or Stenosis | 1 (3.8%) | 1 (6.3%) | 2 (4.8%) |
| Post Liver Transplant | 0 (0.0%) | 1 (6.3%) | 1 (2.4%) |
| Renal Failure | 1 (3.8%) | 2 (12.5%) | 3 (7.1%) |
| Umbilical hernia | 0 (0.0%) | 1 (6.3%) | 1 (2.4%) |
| **Total** | **26 (61.9%)** | **16 (38.1%)** | **42 (100.0%)** |
| **Total** | **437 (60.1%)** | **290 (39.9%)** | **727 (100%)** |
| **Note:** ^a^Neonatal Jaundice, ^b^Cardiogenic Shock, ^c^ Including febrile seizures and status epilepsy | | | |

- Additional File 2, Microsoft Word (.docx), Patient Diagnosis, Table displaying the frequency and percentage of different diagnoses among the neonates and infants.
